# Single-cell morphological tracking of cell states to identify small-molecule modulators of liver differentiation

**DOI:** 10.1016/j.isci.2025.111871

**Published:** 2025-01-23

**Authors:** Rebecca E. Graham, Runshi Zheng, Jesko Wagner, Asier Unciti-Broceta, David C. Hay, Stuart J. Forbes, Victoria L. Gadd, Neil O. Carragher

**Affiliations:** 1 Centre for Clinical Brain Sciences, University of Edinburgh, Edinburgh EH16 4SB, UK; 2 Centre for Regenerative Medicine, Institute of Regeneration and Repair, The University of Edinburgh, Edinburgh EH16 4UU, UK; 3 MRC Human Genetics Unit, Institute of Genetics and Cancer, University of Edinburgh, Edinburgh EH4 2XU, UK; 4 Edinburgh Cancer Research, Institute of Genetics and Cancer, University of Edinburgh, Western General Hospital, Edinburgh EH4 2XU, UK; 5 Cancer Research UK Scotland Centre, Edinburgh EH4 2XU, UK

**Keywords:** Screening in health technology, Biological sciences, Biological sciences research methodologies

## Abstract

We have developed a single-cell assay that combines Cell Painting—a morphological profiling assay—with trajectory inference analysis. We have applied this morphological trajectory inference to the bi-potent HepaRG liver progenitor cell line allowing us to track liver cell fate and map small-molecule-induced changes using a morphological atlas of liver cell differentiation. Our overarching goal is to demonstrate the potential of Cell Painting to study biological processes as continuous trajectories at the single-cell level, enhancing resolution and biological understanding. This work has identified small-molecule Src family kinase inhibitors that promote the differentiation of HepaRG cells toward a hepatocyte-like lineage as well as primary human hepatic progenitor cells toward a hepatocyte-like phenotype *in vitro*. These findings could significantly advance research on liver cell regeneration mechanisms and facilitate the development of cell-based and small-molecule therapies.

## Highlights

• Single-cell trajectory inference applied to image-based Cell Painting assay
• Small-molecule modulators of liver cell differentiation uncovered
• Src family kinase inhibitors promote hepatic liver cell differentiation
• Validated in primary adult human hepatic progenitor cells

## Graphical abstract

**Figure:**
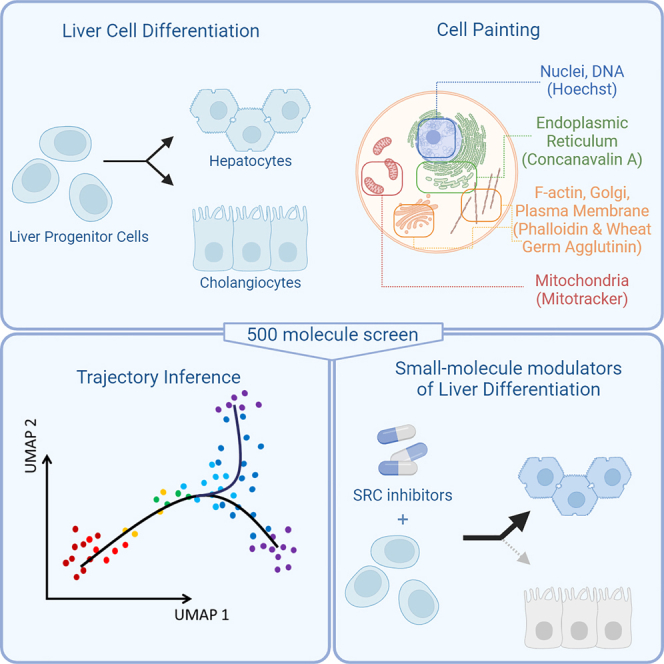

## Introduction

Throughout development, in response to various stimuli, disease onset, and during their lifespan, cells transition between different functional states.^1^ Understanding and controlling cell state transitions and long-term cell fates are crucial for therapeutic developments targeting various developmental disorders and diseases. Key markers of a cell’s physiological state include gene expression and cellular morphology,^2^ providing opportunities for molecular and phenotypic profiling technologies to track cell state transitions and long-term fate decisions. Here, we demonstrate the potential of image-based high-content morphological trajectory inference at the single-cell level in the field of drug discovery.

In this work we have applied this approach to investigate small-molecule modulators of liver cell differentiation. Bi-lineage liver cell differentiation provides an excellent proof of concept example of a continuous, bifurcated, cell state transition. Further this high-content single-cell technique is particularly powerful when applied to multi-cellular assays, as exemplified by liver bi-lineage differentiation as it allows assessment of cell non-autonomous and cell-type specific responses within complex multi-cell assays.

We have used this proof of concept assay to identify small-molecule modulators of liver cell differentiation which have several potential therapeutic benefits. For example, in the liver, hepatocytes are quiescent cells that retain their proliferative capacity*,* allowing for homeostatic and injury-induced liver regeneration.^3^^,^^4^ However, this proliferative capacity is diminished in chronic liver disease. The expansion of hepatic progenitor cells (HPCs) represents an alternative pathway for liver regeneration,^5^^,^^6^ however, the mechanisms and extent of this pathway’s contribution are unclear. Understanding this process and being able to therapeutically manipulate it would be of great benefit since currently the only curative option for patients with severe chronic liver disease is liver transplantation. Unfortunately, donor organ availability falls short of demand; for example, in the UK, approximately 20% of patients die waiting for a donor,^7^ and liver disease accounts for 4% of all deaths (1 in every 25 deaths) worldwide, resulting in two million deaths annually.^8^ Therefore, novel therapeutic strategies are urgently required for the treatment of advanced liver disease.

Both cell-based and small-molecule therapies offer promise as alternatives to whole organ transplants.^9^^,^^10^ However, two major barriers remain; firstly, *in vitro* proliferation of adult human hepatocytes is limited and challenging^11^^,^^12^; second, generating functionally mature liver cell types *in vitro* is only partially successful as primary human hepatocytes rapidly lose most of their differentiated functions.^13^ These challenges limit the application of cell-based therapies and the study of liver cell functions *in vitro*. To overcome these obstacles, our project aims to identify small-molecule modulators that promote the generation of mature liver cell types, aiding cell banking, research into liver cell regeneration mechanisms, and the development of cell-based and small-molecule therapies to treat liver disease.

To track cell state transitions involved in liver cell differentiation, we utilized high-content cellular morphological profiling, an inexpensive, high-throughput, single-cell, spatially resolved alternative to RNA-sequencing for profiling cell states. However, despite collecting single-cell information within each image, most high content imaging data analysis is performed on aggregated cell populations at the “well level”, and single-cell analysis pipelines have not been developed.^14^^,^^15^ In 2013 the Cell Painting assay was developed as an unbiased, cell based, morphological high-content profiling assay that captures subtle changes in morphology.^16^ It combines six fluorescent dyes, imaged in five channels, to visualize eight cellular components and organelles; nuclei, F-actin, endoplasmic reticulum, mitochondria, Golgi apparatus, plasma membrane, cytoplasmic RNA, and nucleoli.^16^^,^^17^^,^^18^ Quantitative data can then be extracted from microscopy images to identify the phenotypic impact of chemical or genetic perturbations. Due to the broad morphological information contained in the images, Cell Painting has been used for various research applications, including clustering chemical and genetic perturbations based on their morphological impact, identifying disease phenotypes, drug screening, toxicity prediction, and mechanism of action prediction using machine learning.^19^^,^^20^^,^^21^^,^^22^^,^^23^^,^^24^^,^^25^

By combining Cell Painting with the bi-potent HepaRG liver progenitor cell line, we have developed the first morphological assay to leverage the mathematics of trajectory inference and apply it to high-dimensional, single-cell morphological data to track liver cell fate. We used this assay to understand how small-molecules modulate liver cell differentiation.

The HepaRG cell line is a popular *in vitro* model and primary human hepatocyte surrogate.^26^ HepaRG progenitor cells demonstrate bi-lineage differentiation potential, producing a mixed population of hepatocyte-like and biliary-like cells over four weeks with commercially available differentiation supplements.^27^ Using the Cell Painting assay to capture morphological changes induced by a bioactivity compound library of 496 target-annotated small-molecules, we identified compounds that induced the same differentiated morphological phenotype as the differentiation supplement when analyzed at the image level by well-level median values. We then developed a single-cell analysis pipeline to track liver cell differentiation using the Cell Painting data and applied trajectory inference to build a single-cell morphological atlas of liver cell differentiation. Finally, we overlaid the drug-induced data onto the morphological atlas to assess how the compounds altered liver cell fates and lineages at the single-cell level.

From this work, we identified compounds and their putative targets and pathways that support expansion and appropriate lineage commitment, aiding the development of cell-based and small-molecule therapies to treat liver disease. We validated our hepatocyte promoting compounds, including Src family kinase (SFK) inhibitors in primary human HPCs, where partial differentiation into hepatocytes was inferred through an increase in hepatic functional markers such as hepatocyte nuclear factor 4 alpha (HNF4a) and cytochrome P450 2E1 (CYP2E1).

Overall, we developed a high-throughput morphological cell state tracking pipeline at the single-cell level to identify small-molecule modulators of liver cell differentiation, which were subsequently validated in lower throughput primary human hepatic progenitor differentiation assays. Single-cell morphological profiling represents a holistic, high-throughput, and inexpensive method to quantify cell state transitions and is applicable to many assay systems, providing deeper insights into the impact of small-molecule perturbations on cellular differentiation.

## Results

### Cell Painting can morphologically distinguish liver cell states

**Figure 1:**
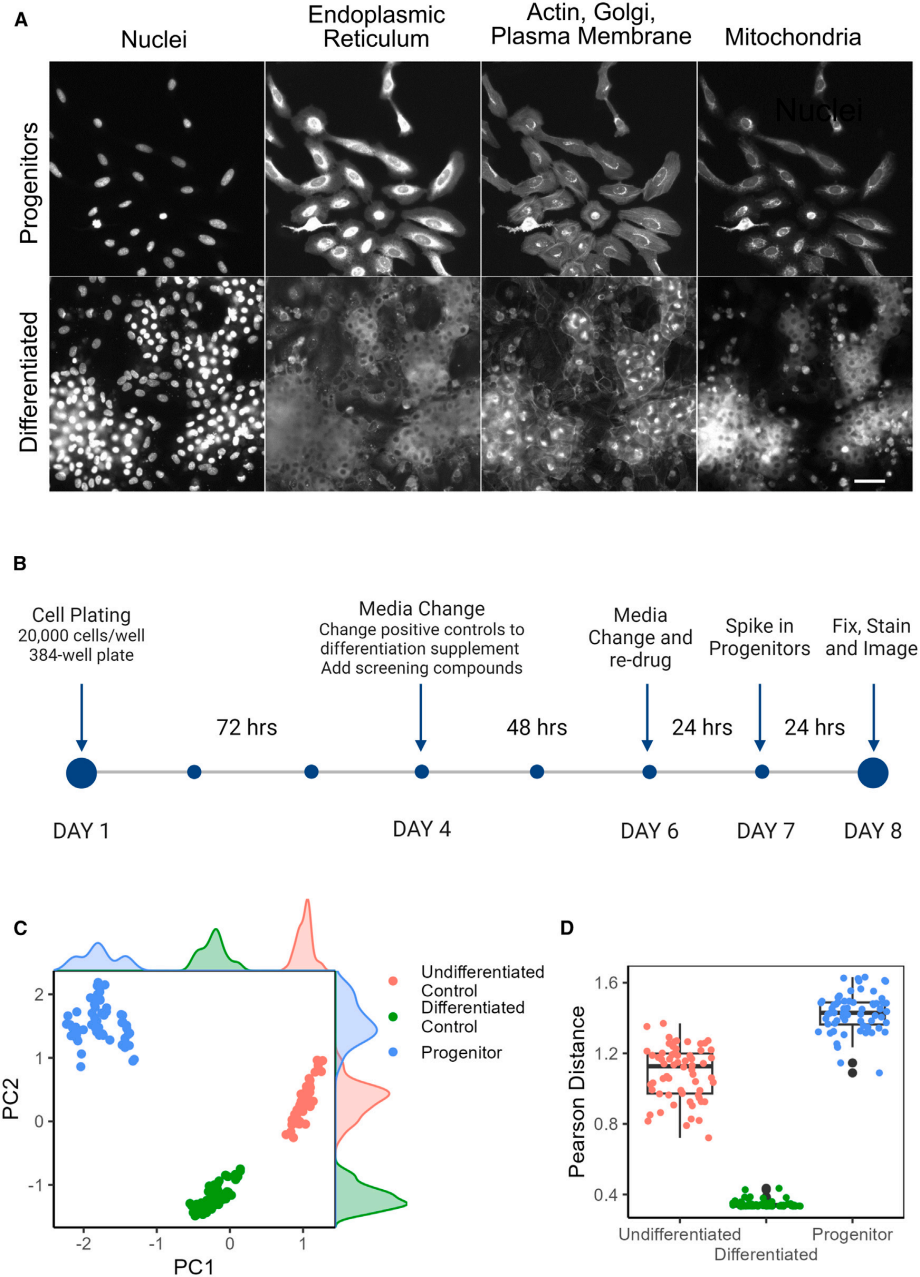
High-content Cell Painting assay in HepaRG cells (A) Cell Painting images of nuclei (Hoechst), endoplasmic reticulum (Concanavalin A), F-actin, golgi and plasma membrane (Phalloidin and Wheatgerm agglutinin), and mitochondria (MitoTracker DeepRed) across HepaRG progenitors and HepaRG differentiated cells. Scale bar, 50 μm. (B) Timeline for 8-day assay. (C) Principal component analysis of assay controls at the image level morphology across 4 replicates. 16 wells of each control per plate and four images per well. (D) Pearson distance from differentiated controls across all control images.

The bi-potent HepaRG liver progenitor cells undergo differentiation to produce a mixed population of biliary-like and hepatocyte-like cells, with distinct morphological changes occurring during this process (Figure 1A). To identify molecules that modulate liver cell differentiation, we developed a high-content morphological assay protocol in 384-well plate format. This protocol is compatible with automated high-throughput imaging and utilizes Cell Painting to track liver cell differentiation in bi-potent HepaRG cells (Figure 1B).

We first assessed the ability of the Cell Painting assay to distinguish HepaRG progenitors from our positive control, HepaRG differentiation medium supplement (differentiated control); and negative control, HepaRG growth medium supplement (undifferentiated control) after eight days in cell culture. Initial analysis was performed using the well-level median morphology metrics, an industry-standard approach for high-content drug screening. After eight days, the differentiated controls were partially differentiated, forming a mix of hepatocyte-like and biliary-like cells (Figure S1). This assay format enables us to identify modulators of liver cell differentiation, including those that enhance differentiation beyond the level of the differentiated controls within the eight-day assay period.

Quantification of the controls was carried out using the median cell value for 848 morphological features extracted with CellProfiler which relate to features across all four channels used in the Cell Painting assay including nuclear, endoplasmic reticulum, actin, Golgi, plasma membrane, and mitochondrial features. The purpose of the Cell Painting assay is that the features are cell type universal and therefore represent an unbiased assay that can be applied to many biological questions. Not all of these features are therefore known to be specifically relevant to liver cell biology but they should capture sufficient information to differentiate between distinct liver cell states to map bilineage differentiation and compound mechanisms-of-action using generic morphology. Principal component analysis and Pearson distance to the positive controls across four separate plate replicates demonstrates clear separation of the controls and reproducible differentiation phenotypes (Figures 1C and 1D). The coefficient of variation (CV) for Pearson distance ranged from 1.2 to 14.6% across the control classes, and plate Z-primes ranged from 0.17 to 0.65 (Figure S2) across four plates. Additionally, a three-class random forest machine learning classifier, trained to distinguish the controls from each other, achieved an accuracy score of 1 (100% accuracy), a precision of 1, a recall of 1 and an F1 score of 1 across the three classes. Together, these results demonstrate a robust assay for drug screening.

### Src family kinases inhibitors modulate HepaRG bilineage differentiation

**Figure 2:**
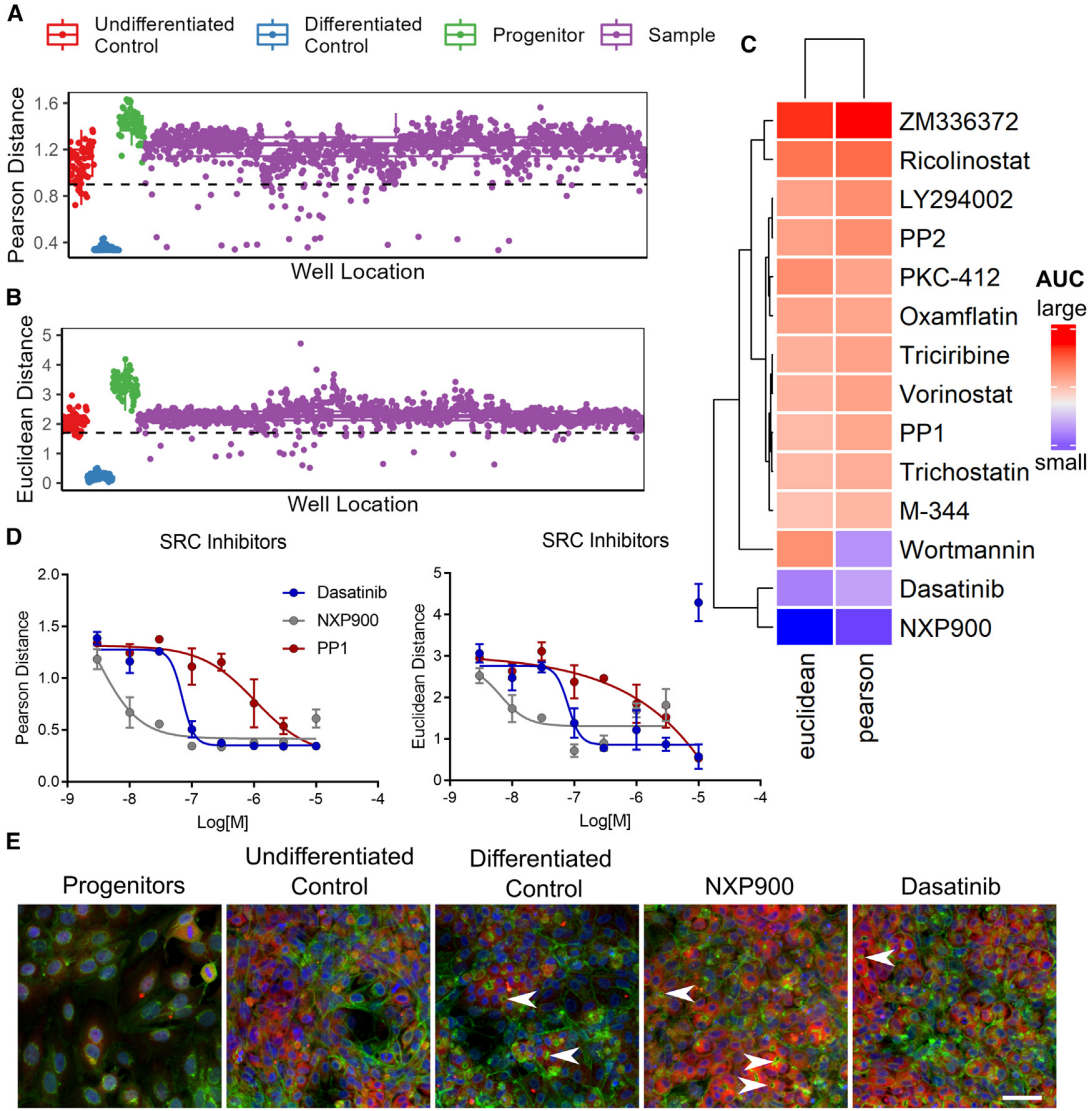
Drug screen analysis (A) Pearson distance and (B) euclidean distance from differentiated controls (blue) across all controls and 496 drug treatments (purple) at 5 and 0.5 μM. Hit thresholds denoted by dashed lines. (C) Area under the curve (AUC) for multiparametric dose responses across 14 compounds of interest. (D) Multiparamentric dose responses across Src family kinase inhibitors for Pearson and Euclidean distance. (Mean ± SD) *n* = 3. (E) Color combined images for controls and top two hits (NXP900 and Dasatinib, 3 μM). Blue = Hoechst (nuclei), Green = Phalloidin and Wheatgerm agglutinin (F-actin, golgi and plasma membrane), Red = Mitotracker DeepRed (Mitochondria). Arrows show canaliculi formation in hepatocyte-like cells. Scale bar, 50 μm.

We used this assay to profile 496 compounds from a library of bioactive target-annotated compounds (Table S1) screened at two concentrations (5 μM and 0.5 μM) to identify hit compounds that promote the differentiation of HepaRG liver cells. We identified 27 hits with a Pearson distance of less than 0.9 to the differentiated control (with differentiation supplement) and a Euclidean distance of less than 1.7 (Figures 2A and 2B). These hits included several classes of compounds, such as histone deacetylase (HDAC) inhibitors, phosphoinositide 3-kinases (PI3K) inhibitors, and SFK inhibitors (Table S2), though we acknowledge that hits may not be acting via their annotated class as several have known polypharmacology. Notably, HDAC inhibitors have previously been shown to promote the differentiation of induced pluripotent stem cells into hepatocyte-like cells,^28^ demonstrating the ability of our HepaRG cell assay to identify biologically relevant small-molecules and pathways.

To confirm the initial screening hits we performed 8-point semi-log dose-response tests on resupplied compound material. Noting that PP1 and PP2 belong to the pyrazolopyrimidine class of cell-permeable compounds known to inhibit SFK members (Lck, Fyn, Hck, and Src) and non-Src family kinases (CSK, RIP2, and CK1δ),^29^ we added two additional SFK inhibitors (dasatinib, a dual Src/Abl inhibitor^30^; and NXP900, a highly selective SFK inhibitor^31^) to determine if PP1 and PP2 were acting via SFK inhibition or other kinase targets. In total we re-tested 14 compounds in triplicate multiparametric dose responses (Figure S3). All compounds demonstrate dose-dependent activity except ZM336372 (Figure 2C). Overall, the most potent hits were the SFK inhibitors, all of which validated, with the most selective SFK inhibitor, NXP900, being the most potent hit with an IC50 of 3nM (Figures 2C–2E).

### A single-cell morphological atlas of HepaRG bi-lineage differentiation

To gain insights at the single-cell level and understand how the compound hits modulate cell fate toward the two different cell lineages, we built a single-cell morphological atlas of HepaRG bilineage differentiation. We then overlaid the single-cell drug-induced changes on to the morphological atlas to quantify how the drugs affected bilineage differentiation using trajectory inference.

**Figure 3:**
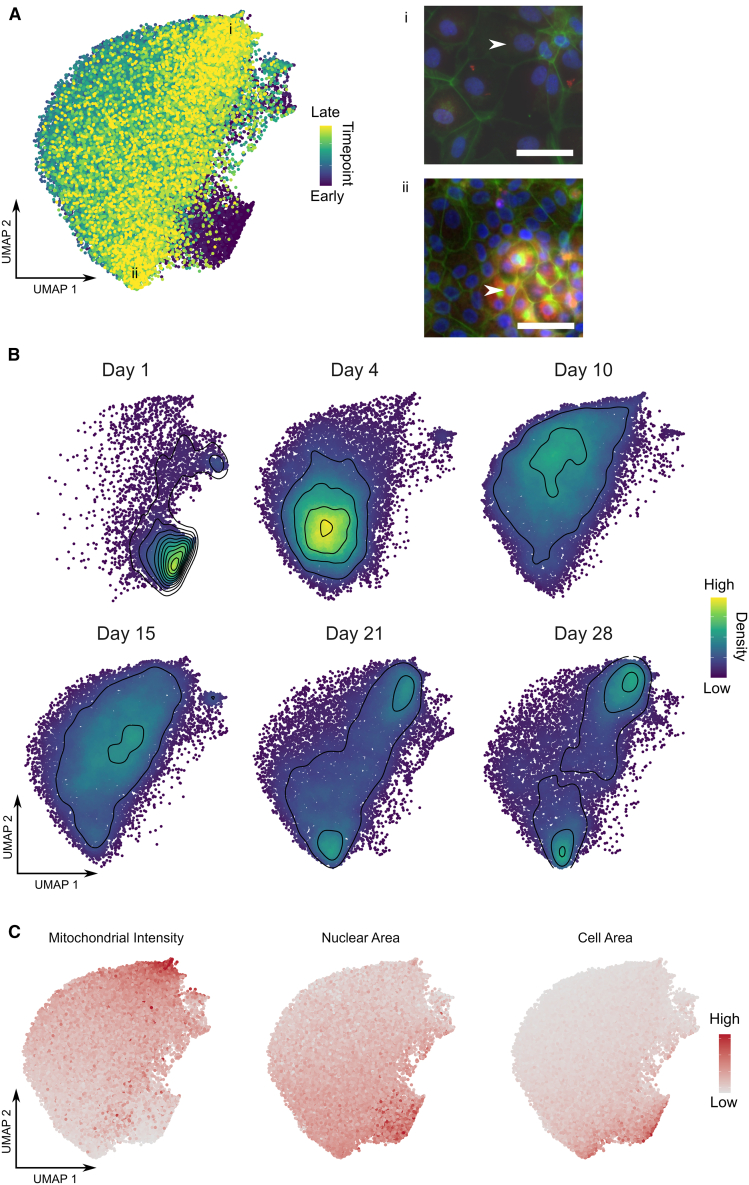
Single cell morphological atlas of HepaRG differentiation (A) Uniform manifold approximation and projection (UMAP) embeddings of single-cell HepaRG time course Cell Painting data colored by the time point at which the cell was imaged. Exemplar colour-combined images of single-cells (white arrows) belonging to the two clusters of differentiated, (final time point) cells (i and ii). Blue = Hoechst (nuclei), Green = Phalloidin and Wheatgerm agglutinin (F-actin, golgi and plasma membrane), Red = Mitotracker DeepRed (Mitochondria). Scale bar, 50 μm. (B) UMAP embeddings separated out across 6 timepoints spanning the differentiation period. Points colored by density on plot and density contours added. (C) UMAP embedding for complete time course data (as in A) colored by characteristic morphological features delineating the hepatocyte-like cells, biliary-like cells and progenitors.

To build the morphological atlas of HepaRG differentiation, we captured Cell Painting images every 24–72 h from 20 time points over the full four-week differentiation protocol, tracking the morphological changes associated with HepaRG bilineage differentiation at the single-cell level (Figure 3). To assess the single-cell data and how the cells morphologically change over time without perturbation, we applied a uniform manifold approximation and projection (UMAP) transform^32^ to the single-cell time course data. This reduced the high-dimensional data down to two components, allowing us to plot the single-cell morphological representations and generate a morphological atlas of untreated bilineage differentiation from progenitors (Day 1) to two distinct final cell populations (Day 28) (Figure 3B).

To confirm that the two final cell populations are the expected clusters of hepatocyte-like and biliary-like cells, we used known cellular morphological markers similar to gene expression markers in single-cell RNA-seq analysis. HepaRG cells robustly produce a 50:50 ratio of hepatocyte-like and biliary-like cells after four weeks, which are easily distinguished under phase contrast due to their distinct morphologies.^33^^,^^34^^,^^35^ It has also been shown that high-content morphological markers such as nuclear size, can separate the hepatocyte and biliary subpopulations of HepaRG cultures, confirmed by cytochrome P450 3A4 (CYP3A4) marker expression.^36^ Here we used cell and nuclear size, as well as mitochondrial intensity, to identify the hepatocyte-like and biliary-like cell populations in the time course data (Figures 1A, 3C, and S4). These markers correspond to the time points at which the cell clusters are expected to appear; progenitors at time point one (Day 1) and the final two clusters of cells at time point 20 (Week 4). The formation of canaliculi structures observed by phalloidin staining is apparent in the identified hepatocyte-like population (Figures 2 and S1), consistent with previous characterization of the cell line.^37^ Overall, the divergence of two separate cell populations marked by expected morphological features is clear, demonstrating the ability of Cell Painting to track a complex process such as bilineage differentiation at the single-cell level.

**Figure 4:**
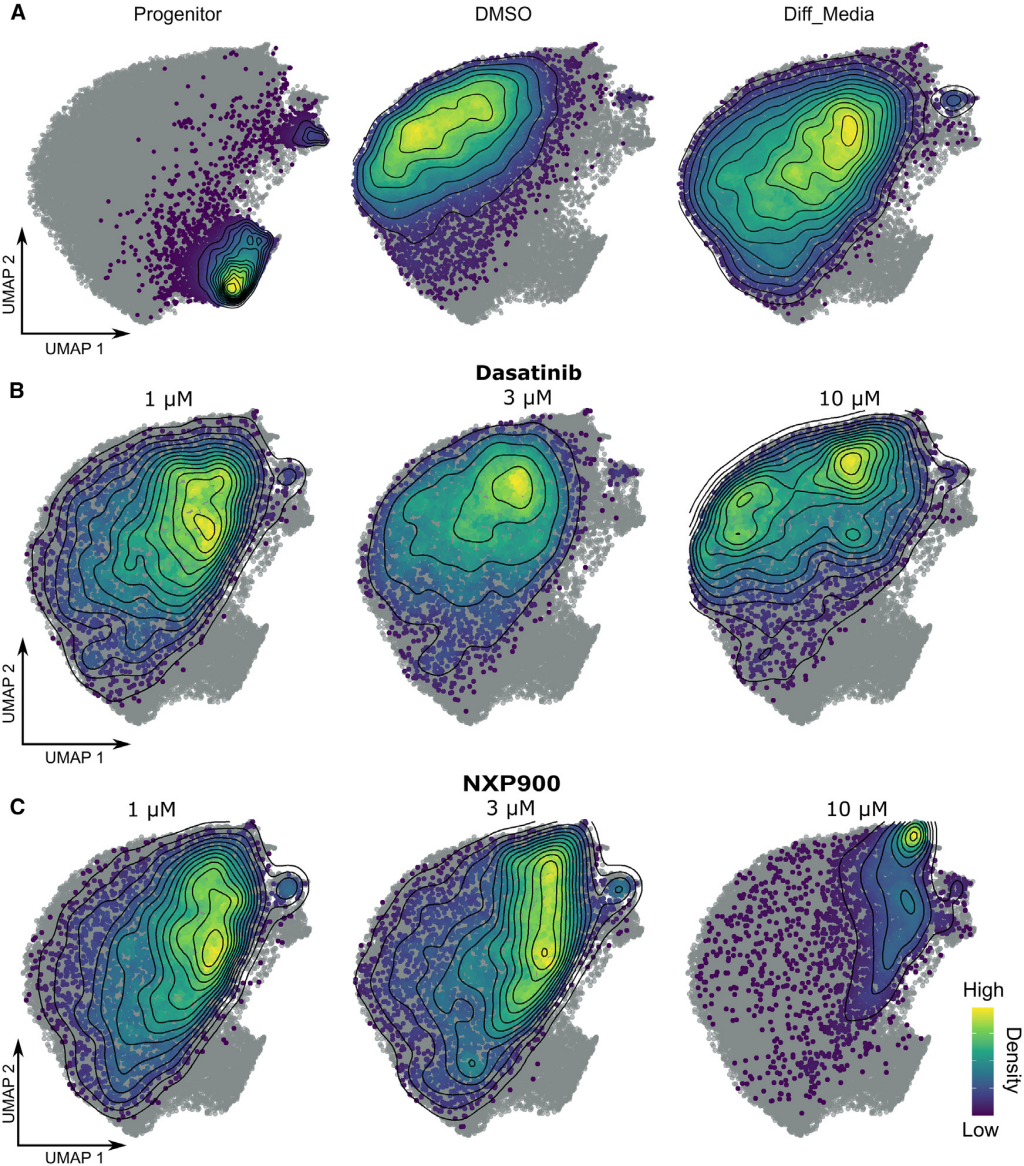
UMAP embeddings for single-cell drug treatments mapped to HepaRG morphological atlas (A) Screening controls, (B) Dasatinib, and (C) NXP900 single-cell data colored by density and overlaid on the HepaRG atlas (gray points). *n* = 3 for drug treatment overlays.

Having confirmed the ability of the assay to resolve the different cell populations and build a continuous morphological atlas of the bilineage differentiation, we next overlaid the small-molecule compound screening data. Firstly, we assessed the effects of the controls at the single-cell level (Figure 4A). Importantly, the “spiked in” progenitor control from the screen (seeded 24 h before the end of the assay) overlaid completely with the original 24-h time point from the time course data used to create the single-cell morphological atlas (Figure 4A), demonstrating the ability to merge datasets at the single-cell level after plate-based standardization to the controls. Secondly, the differentiated control samples in the screening assay pushed the cells closer to the final time points in the time course data compared to the undifferentiated control, although the cells did not reach full maturity (Figure 4A). This was expected since the screening assay was only 8 days long.

**Figure 5:**
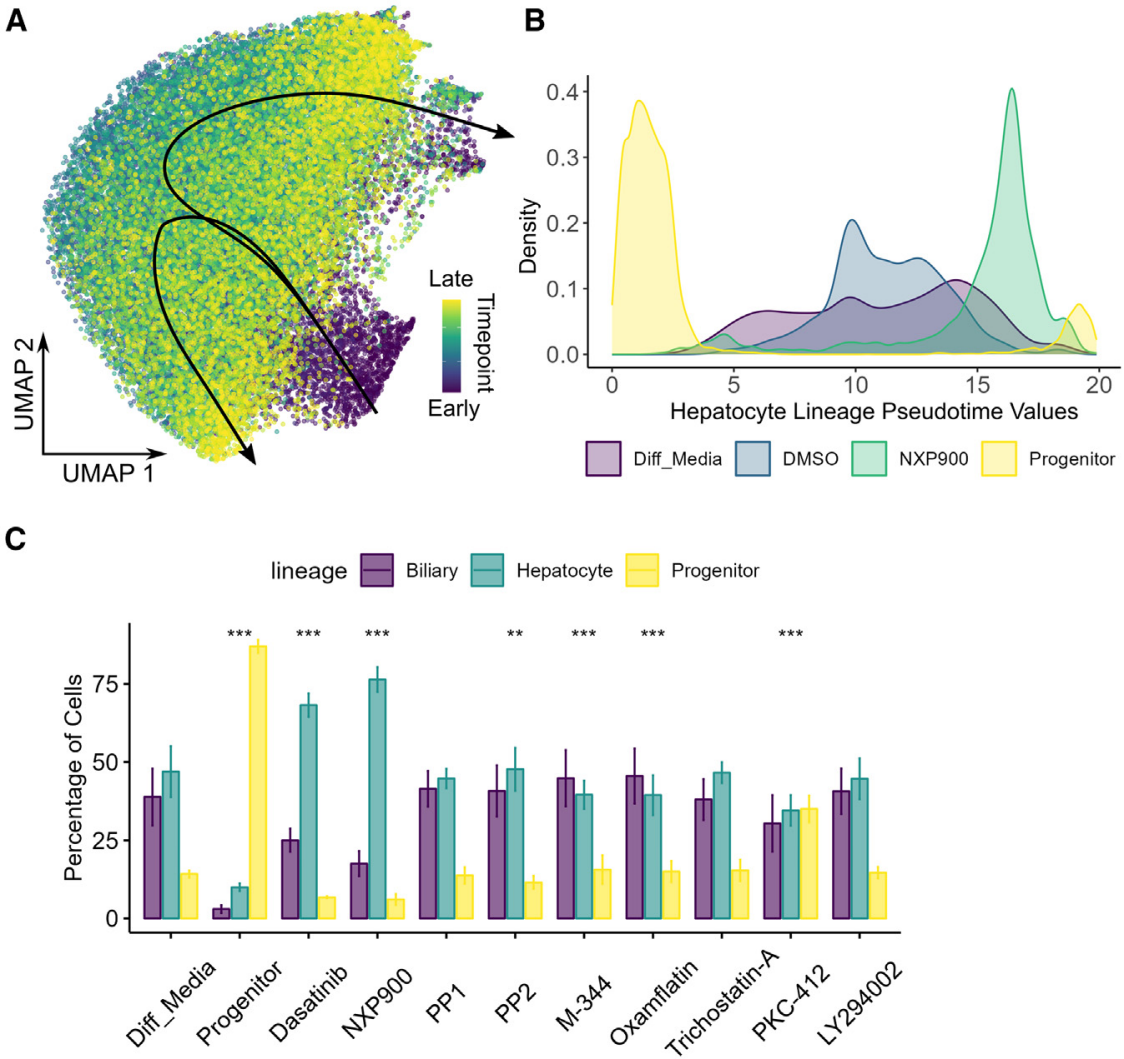
Single-cell morphological trajectory analysis (A) Slingshot trajectory projected onto UMAP embedding of HepaRG differentiation atlas. (B) Density plot showing cell distributions along the hepatocyte lineage trajectory for controls and NXP900 treatment. (C) Percentage of cells belonging to each lineage across compound treatments. (Mean ± SD). Chi square test, reference distribution = differentiated control, ∗∗*p* < 0.01, ∗∗∗*p* < 0.001, *n* = 3.

Next, we overlaid the single-cell data for compound treatments of interest at three concentrations (1, 3, and 10 μM). The confirmed compound treatments show dose-dependent shifts in the cell populations (Figures 5B, 5C, and S6–S8). Consistent with the image-level analysis, the SFK inhibitors demonstrated the greatest effects (Figures 4B and 4C), with the selective SFK inhibitor NXP900 being most potent.

Strikingly in the image-level analysis, the top concentration of NXP900 (10 μM) appears to deviate from the desired phenotype since the Euclidean distance to the positive control increased (Figure 2D). In multiparametric high-content phenotypic profiling studies, such deviations at high concentrations are common and are often thought to be due to cytotoxicity. However, the single-cell analysis revealed that this was because NXP900 treatment had dramatically altered the cell type composition, highly enriching the hepatocyte-like population (Figure 4C). This highlights the additional information and resolution achievable when using the single-cell level data in comparison to the industry-standard image-level analysis of drug treatments. Furthermore, the SFK inhibitor NXP900, and to a certain extent Dasatinib, appeared to promote hepatic differentiation in the eight-day assay to the same extent as the final time points in the time course data when the cells have had the full four weeks to differentiate, therefore outperforming the differentiated controls in the assay, indicating that the SFK inhibitors significantly accelerate the hepatocyte differentiation process.

### Src family kinase inhibitors alter differentiation trajectories of liver cell types

In order to quantify biological progression through this process of bilineage differentiation, we computationally inferred the differentiation trajectory of liver cells from the single-cell Cell Painting atlas by pseudotime mapping. Pseudotime defines the position of the cells along the trajectory and quantifies the relative progression through the underlying biological process being investigated.^38^ For pseudotime mapping we used the slingshot method.^39^ Slingshot trajectory inference was chosen for the single-cell analysis for its applicability to morphological data since it does not have underlying assumptions that would be violated by the data. Further, it does not assume knowledge of the number of differentiation trajectories and does not restrict choice of dimensionality reduction methods. Despite its flexibility, slingshot has been shown to capture a variety of trajectory structures with high accuracy.^40^ Slingshot analysis produced a two lineage, branched trajectory starting from the single-cell progenitor population as expected (Figure 5A). We then projected the compound treated single-cell data onto the liver differentiation trajectories to determine the pseudotime scores and quantitatively compare how the compounds affected the ratios of the two mature cell types using the lineage weightings to assign each cell to either the progenitor (pre-branching of the two lineages), the hepatocyte, or the biliary lineage. From this we can see that the majority of the progenitor control cells map to the portion of the trajectory that is pre-branching (progenitors), while the differentiated control and most of the hit compounds lead to a nearly 50:50 split of the biliary and hepatocyte lineages (Figure 5C). Most of the treatments overlaid similarly to the differentiation control, producing a mixed population of hepatocyte-like and biliary-like cells (Figure 5C). Interestingly, both the approved SFK inhibitor Dasatinib and particularly NXP900 cause a significant increase in cells along the hepatic lineage, increasing the proportion to over 75% in the NXP900 treated populations (Figures 5C and S8). This could have important implications in minimizing heterogeneity *in vitro* and enabling more efficient engraftment *in vivo*.

Lastly, quantification of NXP900 effect led to much greater pseudotime values for the hepatocyte lineage when compared to both the undifferentiated control and the differentiated control (commercially available supplement, see STAR Methods) (Figure 5). This supports the idea that NXP900 promotes the hepatocyte-like cells in the HepaRG line, and further, NXP900 accelerates the differentiation of the hepatocyte-like cells compared to the differentiated control (Figures 4 and 5B) within the eight-day assay window.

### Src family kinase inhibitors promote expression of hepatic markers in primary human hepatic progenitors

**Figure 6:**
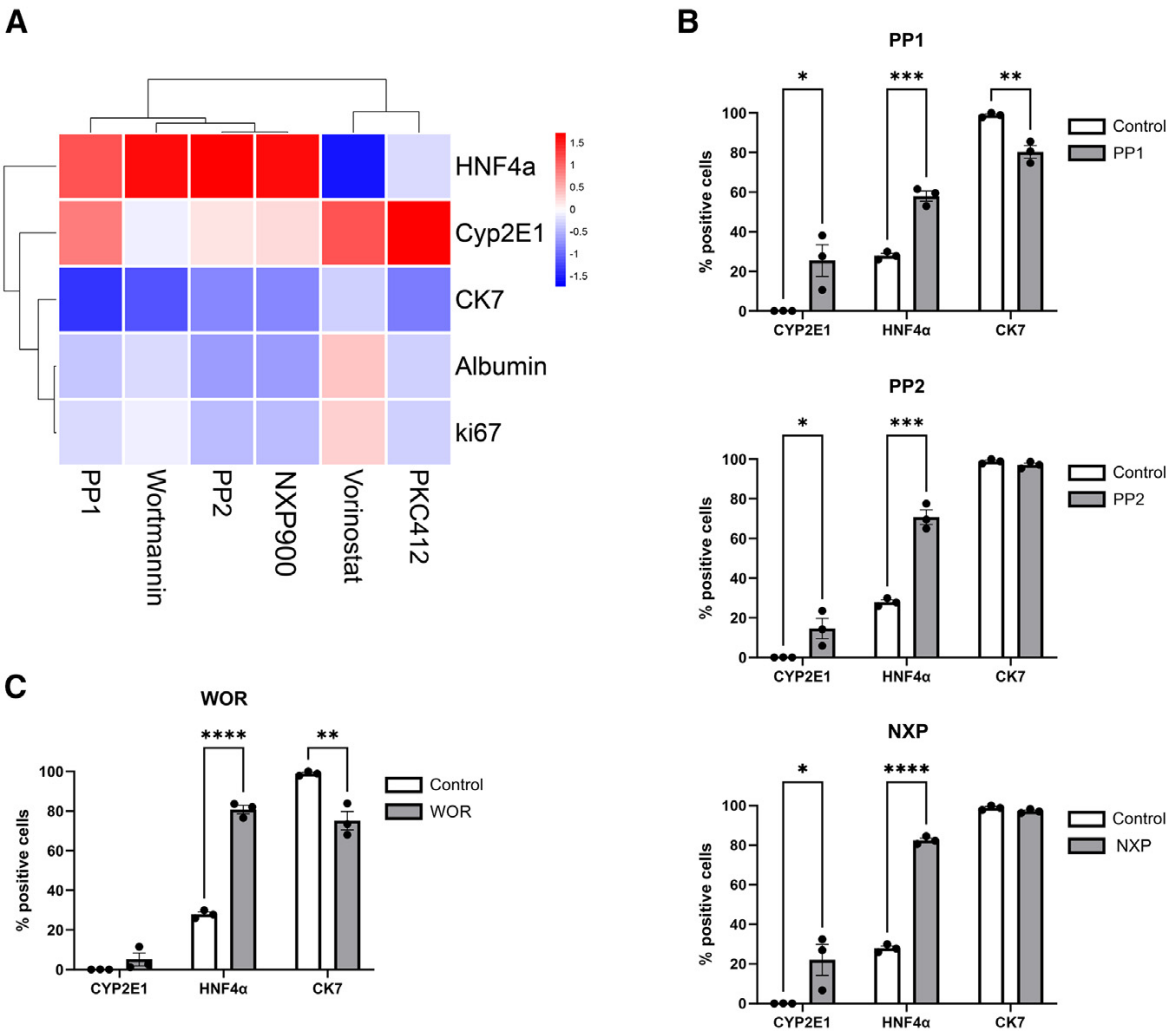
Validation of candidate hepatocyte-like inducing small molecules in primary human HPC (A) Heatmap of phenotypic marker expression in response to compound treatments. Data normalized to control expression. (B) Percentage of cells positive for hepatocyte markers CYP2E1 and HNF4a and biliary epithelial marker CK7 in response to SFK inhibitors. (Mean ± SEM) Multiple t-tests, ∗*p* < 0.05, ∗∗*p* < 0.01, ∗∗∗*p* < 0.001, *n* = 3 per group. (C) Percentage of cells positive for hepatocyte markers CYP2E1 and HNF4a and biliary epithelial marker CK7 in response to PI3K-AKT inhibitor, Wortmannin. (Mean ± SEM) Multiple t-tests, ∗∗*p* < 0.01, ∗∗∗∗*p* < 0.001, *n* = 3 per group.

Our morphological single-cell trajectory inference of small molecule compound perturbations suggests that SFK inhibitors promote the hepatocyte-like lineage in the HepaRG cells, something that cannot be detected using the image level data. Small-molecules that can be added to culture media as a supplement to promote the functional maturity of primary hepatocytes *in vitro* are of great interest to the liver regeneration community. We therefore chose to test the SFK inhibitors in primary adult human HPC, bipotent cells isolated from discarded donor organs, to measure multiple functional hepatic markers and confirm the ability of the compounds to induce differentiation of human HPC toward a hepatocyte-like phenotype. The following markers, HNF4a (an essential transcription factor involved in the functional differentiation of hepatocytes during development), CYP2E1 (cytochrome P450 enzyme) and albumin (essential role in transportation of hydrophobic molecules) were selected to infer hepatocyte differentiation. Biliary epithelial cell marker CK7, and proliferation marker Ki67 were also included in the phenotypic panel. Incubation of human HPC with the SFK inhibitors NXP900, PP1, and PP2 all demonstrate a significant increase in the proportion of cells expressing CYP2E1 and HNF4a compared to untreated controls. Albumin expression remained low across all compound treatments, suggesting only partial differentiation at this time point. Of note, PP1 also significantly reduced the biliary epithelial marker CK7, suggestive of a phenotypic shift (Figures 6A and 6B).

We further tested several other hits from the HepaRG screen in primary human HPC and found that several hits increased a single hepatocyte functional marker (Figure 6A). Interestingly, the PI3K-AKT inhibitor, Wortmannin demonstrated a 40% increase in HNF4a expression coupled with a reduction in CK7 expression (Figure 6C), suggestive of differentiation.

Taken together, these data validate the use of single-cell morphological trajectory inference to identify candidate small-molecule modulators of liver cell differentiation for further testing in primary human cells. Of particular interest would be the use of SFK inhibitor NXP900 as well as inhibitors of the PI3K-AKT pathway.

## Discussion

Understanding and controlling cell state transitions and long-term cell fates are crucial for therapeutic development targeting various developmental disorders and diseases. Key markers of a cell’s physiological state include gene expression and cellular morphology,^2^ providing opportunities for molecular and phenotypic profiling technologies to track cell state transitions and long-term fate decisions.

Single-cell genomic technologies, such as single-cell RNA-sequencing (scRNA-seq),^41^ have significantly advanced in-depth mechanistic studies of cell fate and dynamic cell state transitions.^42^^,^^43^ Recent advances in scRNA-seq and computational trajectory inference methods have been applied to infer the dynamics of cell fate across complex biological models.^39^^,^^44^^,^^45^^,^^46^^,^^47^ Such studies include mapping gene expression and cell phenotypes across the entire mammalian liver^48^ and understanding the progression of liver diseases such as cirrhosis^49^ and non-alcoholic steatohepatitis (NASH).^50^ However, the high-throughput application of scRNA-seq is restricted by cost, limiting its use in early-stage drug discovery for target identification and proof-of-concept studies. In contrast, multiparametric high-content imaging technology is more cost-effective and can generate single-cell morphological phenotypic fingerprints at scale.^51^ Thus, high-content morphological profiling is applicable to systematic phenotypic screening of large chemical libraries and gene targeting libraries, enabling a comprehensive survey of modulators of cell states.

In the work presented here, we started by assessing whether Cell Painting could be used to distinguish liver cell states at the image level. This was carried out using four biological batches of the HepaRG cell line, and 64 images per cell state. Random forest was chosen because there is a reasonably limited number of samples and random forests are less likely to overfit than other models because they are made up of many weak classifiers that are trained independently on completely different subsets of the data. To further avoid overfitting the accuracy reported in the manuscript is based on unseen images, not used in the training of the classifier. The accuracy of 100% is very high, but not necessarily unexpected given the very distinct clustering of the images in principle component space (Figure 1). However, given the small sample size it is possible that our model is overfitting and increasing the number of images it is trained on could reduce this. We further emphasize that this classifier was only used to assess the assay quality since standard assay metrics like Z-Primes are not relevant to multiparametric high-content assays, the random forest classifier was not used to identify hit compounds.

The morphological trajectory inference described here offers a holistic, high-throughput, and cost-effective alternative to antibody-based functional hepatocyte assays and RNA-sequencing. It provides deep insights into the impact of small-molecule perturbations on liver cell differentiation. By enabling the computational alignment and modeling of cellular states, this single-cell Cell Painting analysis pipeline has broad applications, offering a way to quantify single-cell responses across perturbation screens, disease progression, and cellular differentiation. This high-content single-cell technique is particularly powerful when applied to multi-cellular assays, as it allows assessment of cell non-autonomous or cell-type specific responses within complex multi-cell assays. Furthermore, modeling biology as a continuous trajectory enables us to identify off-target and unwanted trajectories caused by compounds, beyond merely assessing perturbations in terms of distance to static phenotypes of interest. This high-throughput compatible assay has exceptional resolution for identifying cell-type specific and biologically relevant hits, and for unraveling cell state transitions, cell dynamics, and lineage specificity.

By building a morphological atlas of HepaRG bilineage differentiation, we could map and quantify how hit compounds affected liver cell differentiation across the two cell lineages. Without the single-cell analysis, we could not have characterized the effects of the drugs on both the hepatocyte and biliary populations within the images given that it is a mixed population and the two cell types cannot be isolated and studied separately in the HepaRG line. The single-cell analysis led to the identification of SFK inhibitors that promote differentiation of liver progenitor cells toward a hepatocyte-like phenotype *in vitro* across HepaRG liver progenitor cells and primary human HPC. NXP900 was shown to increase the hepatic-like population of HepaRG cells to over 75% using high-content morphological markers and pseudotime trajectory analysis. This finding was confirmed in primary human HPC using immunostaining for the hepatic markers HNF4a and CYP2E1. This enrichment for hepatic cells is useful as it minimizes heterogeneity which can impact negatively on *in vitro* model performance. It could also reduce asynchrony in the differentiation process, important in both *in vitro* and *in vivo* contexts, and may enhance cell transplant efficiency, cell engraftment, and tissue integration, thereby boosting hepato-cellular function and improving host liver performance. In future, this assay could be used to find the optimal concentration of compounds for *in vivo* proof-of-concept studies by balancing hepatocyte-induction with minimal toxicity, through measuring total cell number of each cell type population following compound treatment.

While PP1, PP2, and dasatinib show polypharmacology, NXP900 is a potent and selective SFK inhibitor.^52^ We believe the fact that all these SFK inhibitors promote differentiation suggests that it is inhibition of the Src kinase family that is driving the effect and the promiscuity of the other drugs is not having significant influence. However, we cannot rule out polypharmacology or off-target activity. This will require further work in future to experimentally confirm a role for Src and other SFK members in liver bilineage differentiation, and in particular hepatocyte differentiation.

SFK activity and downstream signaling have been implicated in various liver disorders, including hepatocellular carcinoma^53^ and liver fibrosis.^54^ In addition, a role for c-Src in cell fate determination during endodermal commitment of human iPSCs has been described,^55^ with the kinase inhibitor PP2 shown to protect against keratin mutation-induced mouse liver injury through Src inhibition.^56^ Previous studies have demonstrated that genetic deletion of Src’s binding partner, focal adhesion kinase (FAK), accelerates liver regeneration.^57^ Our study is the first to demonstrate a role for small-molecule SFK inhibitors in promoting hepatocyte progenitor differentiation, presenting a potential therapeutic opportunity for liver regeneration.

Unlike currently approved dual Src-Abl inhibitors, including dasatinib, which block Src in the active “open” conformation,^58^ NXP900 binds and locks Src tyrosine kinase in a closed inactive conformation, inhibiting both scaffolding and catalytic activity.^52^ This mechanism of action contributes to NXP900’s unique target selectivity profile, with 1000-fold selectivity for SFK members over Abl kinase, resulting in highly potent, selective, and sustained pathway inhibition *in vitro* and *in vivo.*^52^ This likely explains NXP900’s higher potency compared to other SFK inhibitors. With dasatinib already FDA-approved and NXP900 in phase 1 clinical studies, both represent promising drug candidates to stimulate liver cell differentiation *in situ*, potentially restoring damaged liver function. SFK inhibitors could also be used as cell culture additives to maintain liver differentiation *in vitro* for biological investigation and drug discovery applications. While, NXP900 has an IC50 of 0.5 nM against Src in biochemical assays, in cell assays complete inhibition of phospho Src is achieved only at 100 nM concentrations or above and it is known that 1–10 μM is still a dose range in which there is complete Src inhibition with minimal effects on other kinases.^52^ While we cannot rule out off-target effects, these results combined with the identification of other structurally distinct Src inhibitors among the hits in our assay (Figure 2) strongly indicate that NXP900 is inducing hepatocyte differentiation through inhibition of the Src family kinases.

In summary, we describe the application of trajectory inference to high-throughput morphological data to quantify cell state transitions at the single-cell level in a high-throughput screening format. We demonstrated the utility of this method by screening a small-molecule compound library in a liver progenitor differentiation assay and validated the hits in primary human hepatic progenitor differentiation assays. Our assay is validated in a high-throughput format and suitable for screening larger libraries of target-annotated compounds and diverse chemicals to identify additional therapeutic targets and chemical starting points, launching new drug discovery programs for liver regeneration. The validated hits from our pilot screen include SFK inhibitors, revealing a role for Src and other SFK members in accelerating hepatocyte differentiation. Future work includes *in vitro* functional assays such as drug metabolism and albumin secretion, and *in vivo* studies to investigate the effects on the expansion and differentiation of exogenously applied hepatocytes and the ability to target endogenous HPC repair mechanisms. Single-cell morphological trajectory inference is applicable to other high-content image-based assays, particularly *in vitro* stem cell differentiation and disease progression models, offering broad utility for investigating modulators of cell state transitions.

### Limitations of the study

Due to the size of single cell datasets and the computational power required to work with them the scale of this study was relatively small for high-throughput drug screens. Based on our pilot study, large scale screens are feasible but will have computational infrastructure requirements and will require support from dedicated bioinformaticians until open-source tools for single-cell morphological analysis are fully developed. Specifically, we feel that application across larger sets of target annotated and diverse chemical libraries would fully determine the utility of the single-cell morphological analysis methods described in this paper to identify new targets and chemical starting points for new drug discovery programs targeting liver disease.

Secondly, due to limited availability of primary human hepatocyte progenitor cells, hit compounds were not tested as full dose responses in the secondary assay, which would be informative in future work. Further, a more in-depth analysis of drug mechanism at the molecular level across a greater diversity of hepatocyte progenitor cell lines incorporating gender, ethnicity, and lifestyle differences would be informative in disease positioning of potential drug candidates for clinical studies, however, in our study this was limited due to the availability of discarded donor organs.

Lastly, in this work we identified several SFK inhibitors as modulators of liver cell differentiation. In humans, there are nine SFK members including Src, Yes, Hck, Lck, Fyn, Fgr, Lyn, Yrk, and Blk. Systematic genetic knockdown studies using CRISPR technologies would help identify which specific SFK members are contributing to the positive effects of the SFK inhibitors identified in this study.

## Resource availability

### Lead contact

Further information and requests for resources and reagents should be directed to and will be fulfilled by the lead contact, Rebecca E. Graham (r.graham@ed.ac.uk).

### Materials availability

This study did not generate new unique reagents.

### Data and code availability

• Raw data from Figures 1, 2, 3, 4, and 5 have been deposited at Mendeley and are publicly available as of the date of publication at (Mendeley Data: https://doi.org/10.17632/8r8r5vvc3p.1). The DOI is also listed in the key resources table.
• All original code has been deposited at Zenodo and is publicly available at (https://doi.org/10.5281/zenodo.14646094) as of the date of publication. Its also available at https://github.com/CarragherLab/sc_morph_tracking.
• Any additional information required to reanalyse the data reported in this paper is available from the lead contact upon request.

## Acknowledgments

This work was supported by the UK Medical Research Council—UK Regenerative Medicine Platform (MR/R015635/1). We thank Justyna Cholewa-Waclaw and the High Content and Imaging core facility at Institute for Regeneration Repair for their technical help with experimental design, data acquisition, and analysis. R.E.G. was funded by MRC Transition Fellowship. The graphical abstract and Figure 1B were created in BioRender, https://BioRender.com/o18t935.

## Author contributions

R.E.G contributed to the conceptualization, acquisition, analysis, and interpretation of the data, as well as drafting the manuscript. R.Z contributed acquisition and analysis of the data. J.W contributed designing the analysis. A.U.-B provided reagents and contributed to interpretation of the data and manuscript revision. D.C.H contributed conceptualization of the work. S.J.F contributed conceptualization of the work as well as interpretation of the data. V.L.G contributed design of the work and acquisition, analysis, and interpretation of the data, as well as drafting the manuscript. N.O.C contributed conceptualization and drafting the manuscript. All authors contributed to the discussion of the results and approved the final version of the manuscript.

## Declaration of interests

A.U.-B and N.O.C disclose the following patents, EP3298015B1, JP6684831B2, US10294227B2, CN107849050B, and CA3021550A1 pertaining to the discovery of eCF506/NXP900 that have been licensed to Nuvectis Pharma Inc. A.U-B and N.O.C hold grants from Nuvectis Pharma to study eCF06/NXP900 outside of the submitted work. D.C.H is a co-founder, director and shareholder in Stimuliver ApS and Stemnovate Limited.

## STAR★Methods

### Key resources table

**Table:** 

REAGENT or RESOURCE	SOURCE	IDENTIFIER
**Antibodies**		

Rabbit anti-CYP2E1	Atlas Antibodies	Cat#HPA009128; RRID: AB_1078613
Rabbit Monoclonal anti-Cytokeratin 7	Abcam	Cat#ab68459; RRID: AB_1139824
Mouse Monoclonal anti-HNF4α	R&D Systems	Cat#PP-H1415-0C; RRID: AB_3659607
Rabbit polyclonal anti-Albumin	Abcam	Cat#ab2406; RRID: AB_303048
Rabbit Recombinant Monoclonal anti-Ki67	Abcam	Cat#ab1667; RRID: AB_302459

**Biological samples**		

Primary human hepatic progenitor cells (HPC)	Derived from donor organs according to Hallett et al.^1^	N/A

**Chemicals, peptides, and recombinant proteins**		

N-acetylcysteine	Sigma-Aldrich	Cat#A9165
Nicotinamide	Sigma-Aldrich	Cat#N0636
Gastrin	Sigma-Aldrich	Cat#G9020
A83-01	Miltenyi Biotec	Cat#130-105-333
Human FGF-10	Peprotech	Cat#100-26-100
Human HGF	Peprotech	Cat#100-39
Human EGF	Peprotech	Cat#AF-100-15-100
Human R-Spondin-1	Peprotech	Cat#120-38
Forskolin	Tocris	Cat#1099
ROCK inhibitor Y-27632	Miltenyi Biotec	Cat#130-103-922
GlutaMAX	Gibco	Cat#35050038
Penicillin/Streptomycin	Gibco	Cat#15140-122
HEPES buffer	Gibco	Cat#15630-056
B27	Gibco	Cat#12587010
N2	Gibco	Cat#17502001
Hoechst 33342	Molecular Probes	Cat#H1399
Phalloidin 594	Abcam	Cat#ab176757
Wheat germ agglutinin Alexa Fluor 594	Invitrogen	Cat#W11262
Concanavalin A Alexa Fluor 488	Invitrogen	Cat#C11252
MitoTracker DeepRed	Invitrogen	Cat#M22426
Cell Mask	ThermoFisher	Cat#C10046
DAPI	Scientific Laboratory Supplies	Cat#D9542

**Critical commercial assays**		

HepaRG® Growth Medium Supplement	Biopredic International	Cat#ADD711C
HepaRG® Differentiation Medium Supplement	Biopredic International	Cat#ADD721C

**Deposited data**		

Raw data and CellProfiler pipeline	This paper	Mendeley Data: https://doi.org/10.17632/8r8r5vvc3p.1

**Experimental models: Cell lines**		

HepaRG® cells	Biopredic International	Cat# HPR101

**Software and algorithms**		

Slingshot	Street et al.^2^	https://github.com/kstreet13/slingshot; RRID: SCR_017012
Mclust R package	Scrucca et al.^3^	https://doi.org/10.32614/CRAN.package.mclust
CellProfiler v4.2.1	Broad Institute	http://cellprofiler.org; RRID: SCR_007358
Harmony	Revvity	
StratomineR	CoreLife Analytics	https://corelifeanalytics.com
RandomForest R package		https://doi.org/10.32614/CRAN.package.randomForest
GraphPad Prism 7	GraphPad	http://www.graphpad.com; RRID: SCR_002798
Code in this study	This paper	https://doi.org/10.5281/zenodo.14646094https://github.com/CarragherLab/sc_morph_tracking

**Other**		

Opera Phenix High Content Screen System	Perkin Elmer	N/A
ImageXpress Confocal	Molecular Devices	N/A
Biomek FX	Beckman Coulter	N/A
Robotic plate loader (Scara4)	PAA	N/A

### Experimental model and subject details

#### Cell lines

HepaRG cells: Human HepaRG cells were supplied by Biopredic International (Cat# HPR101) and cultured in William’s E medium supplemented with GlutaMAX (2 mM) and HepaRG Growth Medium Supplement (Biopredic International, Cat# ADD711C) and differentiated using HepaRG Differentiation Medium Supplement (Biopredic International, Cat# ADD721C) according to the user guide. Briefly, cells were thawed and subcultured to create Master and Working banks of frozen cells. For this, cells were passaged at day 7 post-thaw and subsequently every 14 days for expansion and cryopreservation. Cells were incubated at 37°C, 5% C02 and media was changed every 2–3 days. For differentiation, at day 14 instead of passaging the cells, the media supplement was switched to HepaRG Differentiation Medium Supplement (Biopredic International, Cat# ADD721C) and continued for a further two weeks at which point the cells are fully differentiated.

Primary human hepatic progenitor cells: Cells were isolated from discarded donor organs as previously described.^59^ Briefly, the donor liver is chopped into small pieces before undergoing enzymatic digestion and CD133 selection via magnetic activated cell sorting (Miltenyi Biotec, Cat# 170-076-719). Primary human HPC are routinely expanded as 3D organoids suspended in Matrigel (Corning) and cultured in expansion media; AdDMEM/F12 (Invitrogen) base media, supplemented with Pen-Strep (Invitrogen, 15140-122), HEPES buffer (10mM, Gibco) Glutamax (Life Technologies), B27 (Life Technologies), N2 (Life Technologies), N-acetylcysteine (1.25mM, Sigma-Aldrich), Nicotinamide (10mM, Sigma-Aldrich), gastrin (10nM, Sigma-Aldrich), A83-01 (5μM, Miltenyi Biotec), the growth factors: FGF10 (100 ng/mL, Perprotech), HGF (25 ng/mL, R&D Systems), EGF (50 ng/mL, Peprotech), Rspo1 (500 ng/mL, Peprotech), Forskolin (10μM) and the ROCK inhibitor: Y-27632 (10μM, Miltenyi Biotech). Sample size was one since all screening replicates were derived from the same donor.

### Method details

#### HepaRG assay

##### Compound screening

A library of 496 known bioactive compounds was screened, including kinase, protease, epigenetic libraries, and a subset of FDA/EMA approved drugs (Table S1). Compounds were solubilized in DMSO at 1,000-fold assay concentration in 384-well plates and transferred using a Biomek FX liquid handler, with a final DMSO concentration of 0.1%.

The primary screen was conducted as a single replicate at two compound concentrations (5 μM and 0.5 μM), with the validation 8-point dose-response study performed in triplicate. HepaRG cells (passages 16–18) were seeded at 20,000 cells per well in 384-well plates, incubated for a week with media changes every 2–3 days. After 72 h, plates were treated with compounds for 96 h with a media change and drug re-addition at 48 h. 24 h before the end of the assay, progenitor control HepaRG cells were spiked into an empty column at 3000 cells per well to normalize to the timecourse dataset. Plates were fixed in 4% formaldehyde, stained (see cell painting section below), and imaged on an ImageXpress Confocal (Molecular Devices, USA) equipped with a robotic plate loader (Scara4, PAA, UK) (see image acquisition below).

##### Timecourse study

For untreated timecourse work, cells were seeded at 3,000 cells per well into 16 wells across 20x 384-well plates (Greiner, Cat# 781091), with 70% media changes every 2–3 days. One plate was fixed in 4% formaldehyde every 24–72 h for 28 days (Table S3). For the last two weeks, the supplement was changed to HepaRG Differentiation Medium Supplement, and media changes continued until all plates were fixed.

##### Cell painting

Cell Painting was performed according to established protocols with modifications. Mitotracker DeepRed was added post-fixation. After fixation, plates were washed twice with PBS and staining solution (Table S4) containing 0.1% Triton X-100 in 1% bovine serum albumin (BSA) was added. The Syto14 stain was omitted to reserve the option for future cell type-specific markers. Staining solution was added to each well (25 μL) and incubated in the dark at room temperature for 30 min, followed by three washes with PBS and no final aspiration. Plates were foil sealed.

##### Image acquisition

Plates were imaged on an ImageXpress Confocal (Molecular Devices, USA) equipped with a robotic plate loader (Scara4, PAA, UK). Four fields of view were captured per well using a 20× objective and four filters (Table S4). In total we captured and used 5,120 images for the morphological atlas, 24,576 images for the primary screen and 18,432 images for the validation work.

##### Image analysis

CellProfiler v4.2.1 software was used to segment cells and extract 1004 features per cell per image (pipeline available see key resources table for details). The pipeline identified nuclei from the DAPI channel, using these as seeds to aid a segmentation algorithm to identify cell boundaries from the TxRed channel. These masks were subtracted to give the cytoplasmic region. Morphological features, including size, shape, texture, and intensity, were measured across the image the segmentation masks for the nuclei, cytoplasm, and cells across four image channels. Features therefore include those relating to the nuclei, endoplasmic reticulum, actin, Golgi, plasma membrane, and mitochondria. Median and standard deviation values were calculated for all features per image, resulting in 2008 features total. StratomineR software was used for feature selection, normalization, feature scaling, dimension reduction, distance, and similarity metrics. 848 features were used in the analysis. Images were removed that failed image qc using metrics extracted from CellProfiler. Plates were normalized to the samples on them, and features scaled using robust z-scores. Principal component analysis was applied for dimension reduction, and hits were defined based on Euclidean and Pearson distances.

A three-class Random Forest classifier was implemented on the three control classes (Progenitors, Undifferentiated, Differentiated) using R’s RandomForest package with default parameters. There were 64 data points in each class. The default number of features (square root of the number of features) was used leading to 28 variables at each split, and the default number of 500 trees was used. There were 64 data points in each class, making it a balanced classifier. Validation analysis used median feature values only (not standard deviation features) in the analysis. Plates were normalized to negative controls. Dose-response curves were fitted using GraphPad software (7.05) with the variable slope (four parameters) algorithm.

##### Single-cell analysis

Single-cell datasets were filtered based on the above image qc, and then on single cell nuclear and total cell area parameters, and down-sampled to 15,000 cells per timepoint/treatment. Standardization for the time-course and drug screening data was carried out separately using progenitor controls before combining (788 morphological features). Principal component analysis followed by UMAP on the top 10 components was used for visualizing combined single-cell data. Progenitor cells (24 h post-seeding) overlaid perfectly across datasets, negating the need for single-cell batch correction. A balanced three-class random forest classifier (Progenitor, Doublet, Inlier) was trained to remove doublet cells. The cleaned dataset was reanalyzed.

##### Trajectory inference

Single-cell trajectory analysis was performed using the R package Slingshot. Timecourse and drug-treated data were clustered using Gaussian mixture modeling implemented in the R package mclust.^60^ Trajectories were generated by defining the trajectory backbone by selecting clusters representing progenitor starting cells and the two differentiated cell types. Slingshot^61^ was then employed, using the first two UMAP coordinates, single-cell cluster labels, and the defined trajectory backbone clusters (start and endpoints).

Drug-treated single-cell data (1, 3 and 10 μM) were then projected onto the time-course trajectories. The UMAP embedding of the drug data was used to position the cells along the pre-constructed trajectories using the `predict` function in Slingshot, creating a hybrid object containing the timecourse data trajectories with pseudotime values and weights for the drug-treated cells.

Multiple iterations of the single-cell trajectory analysis produced consistent embeddings, clusterings, and trajectory results. Lineage assignment for single cells was derived from Slingshot lineage weightings. Cells were classified as "Hepatocyte" if they had a lineage 1 weighting of 1, a lineage 2 weighting of less than 1, and a pseudotime value greater than 3 (branching timepoint). "Biliary" labels were assigned to cells with the opposite lineage weightings, while the "Progenitor" label was assigned to cells with a pseudotime value of 3 or less.

#### Primary human HPC differentiation assay

##### Cell plating

Prior to compound screening, 3D organoids were dissociated into single cells and seeded on laminin-521 coated plates to expand as 2D cells. Organoids were washed in cold PBS-EDTA (PE; 10mM, Gibco), broken down with 1U/mL Dispase for 30 min at 37°C, further disrupted via manual pipetting, and incubated for an additional 30 min. The resulting cells were washed with cold PE and treated with a TrypLE mix solution (10X TrypLE + 1X TrypLE at 1:4) for 5 min with gentle agitation at 37°C to obtain single cells. Cells were plated on laminin-521 (10 μg/mL) coated plates and cultured in expansion media, which was replenished every 3–4 days.

##### Compound screening

2D human HPCs were seeded into 96-well plates (2 x 10ˆ4 cells/well) and allowed to attach for 24 h. The compounds and their final concentrations used for validation are listed in Table S5. Selected compounds were administered with fresh expansion media every 48 h. After 7 days, cells were immunostained for various phenotypic markers and counterstained with Cell Mask Deep Red (cell membrane) and DAPI (nuclei) (Table S6).

##### Immunocytochemistry

Human HPCs were fixed with 1:1 methanol-acetone or 10% formalin for 20 min. Cells were permeabilized with 0.1% Triton X-100 (Sigma) in PBS for 15 min, followed by a protein block (Abcam) for 30 min. Cells were then incubated with relevant primary antibodies diluted in antibody diluent (Abcam) at 4°C overnight. Finally, cells were stained with Alexa Fluor 488 secondary antibodies (Invitrogen), combined with Cell Mask and DAPI, for 30 min at room temperature.

##### Image acquisition and analysis

Plates were imaged using the Opera Phenix High Content Screen System and analyzed with Harmony software. Total cell numbers were determined by detecting cell membranes and nuclei. Cells positive for specific phenotypic markers were identified using expression thresholds over relevant isotype controls and expressed as a percentage of positive cells per well. Experiments performed in triplicate.

### Quantification and statistical analysis

Statistical details including the tests used can be found in the figure legends. The ‘slice’ function in R was used to down sample cells per group. Pearson and Euclidean dose-response curves were fitted using GraphPad software (7.05) with the variable slope (four parameters) algorithm. Wilcoxon rank sum (Mann-Whitney test) was used after Shapiro-Wilk’s normality test for the Cell Painting data. T-tests were used for the human HPC experiments. Significance was defined as *p* < 0.05.

## Supplemental information

Document S1. Figures S1–S8 and Tables S2–S6

Table S1. Chemical SMILES for bioactive compound library screened in HepaRG cells
