# Supplementary material for: Single-cell morphological tracking of cell states to identify small-molecule modulators of liver differentiation
Source: iScience. 2025 Jan 23;28(2):111871. doi: 10.1016/j.isci.2025.111871 (PMC11848441; doi:10.1016/j.isci.2025.111871)
Supplement: Document S1. Figures S1–S8 and Tables S2–S6 [file mmc1.pdf]

iScience, Volume *28*

Supplemental information

Single-cell morphological tracking of cell states to identify small-molecule modulators of liver differentiation

Rebecca E. Graham, Runshi Zheng, Jesko Wagner, Asier Unciti-Broceta, David C. Hay, Stuart J. Forbes, Victoria L. Gadd, and Neil O. Carragher

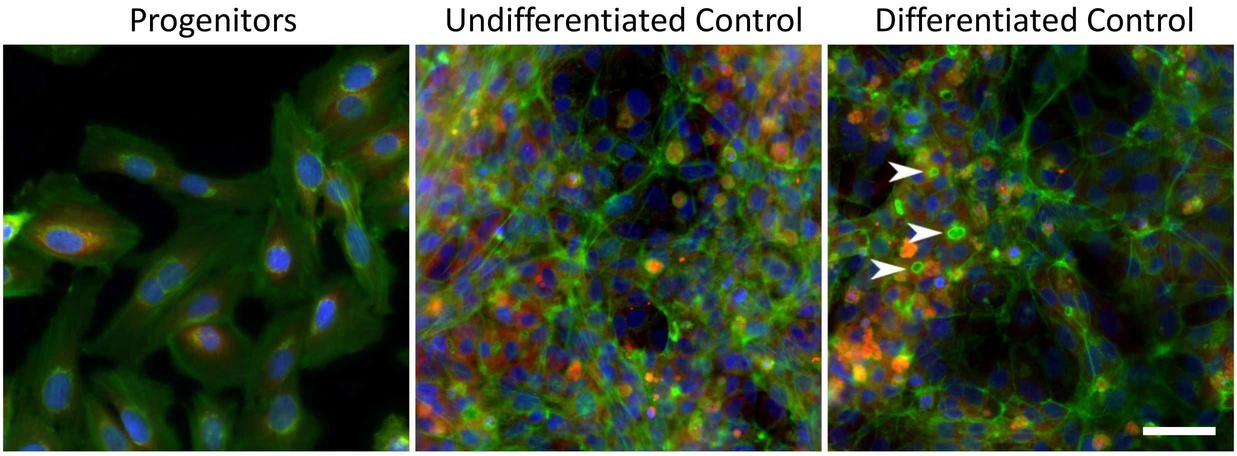

Figure S1. Exemplar colour combined images of assay control HepaRG cell populations, Related to Figure 1. Blue = Hoechst, Green = Phalloidin and Wheatgerm Agglutinin, Red = Mitotracker DeepRed. Arrows show canaliculi formation in hepatocyte-like cells. Scale bar = 50µm

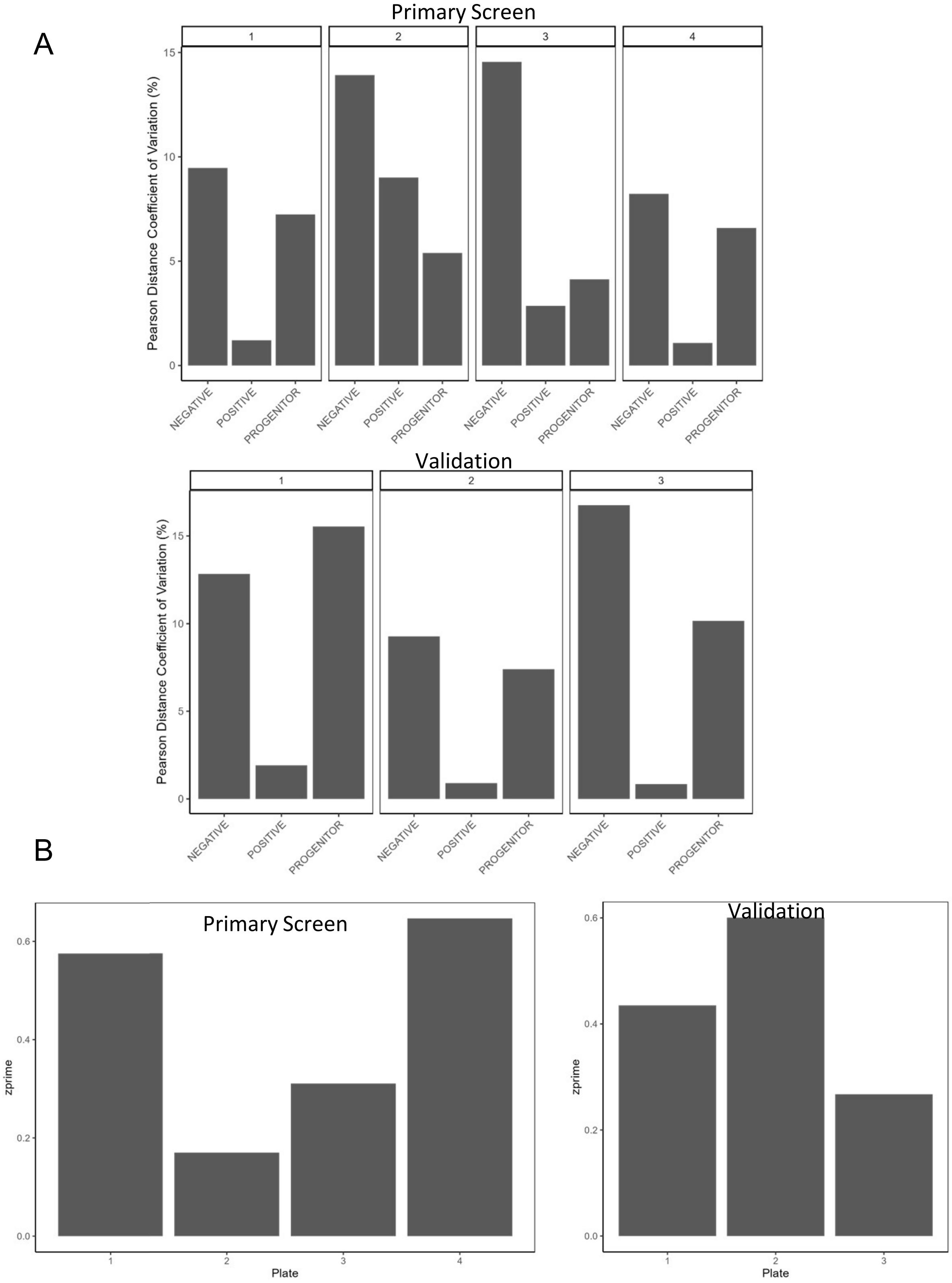

Figure S2. Assay performance metrics, related to Figure 2. A) Coefficient of variation for Pearson distance across plates and control classes for primary and validation plates. B) Plate Z-primes across primary and validation plates.

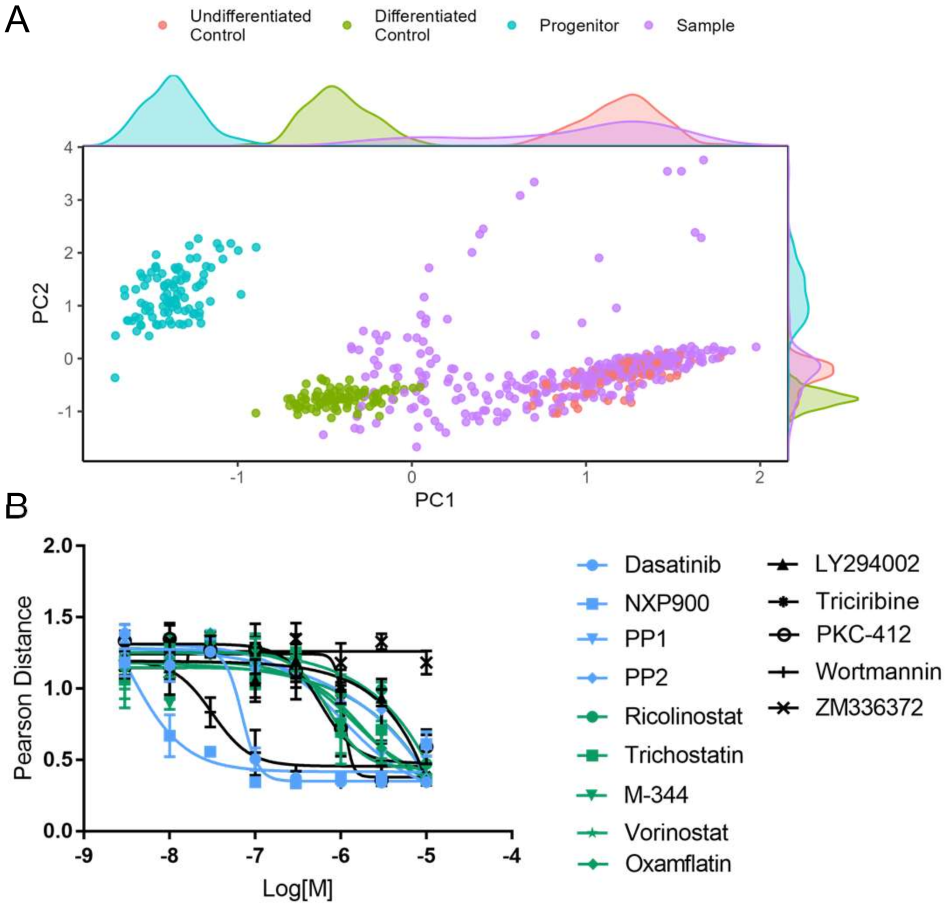

Figure S3. Image level validation data, related to Figure 2. A) Principal component analysis of image level morphological changes caused by 14 validation drugs in dose response (purple) and controls. n = 3. B) Multiparametric dose response analysis for the 14 validation compounds using Pearson distance to the Differentiated controls. Blue = Src family kinase inhibitor, green = Histone deacetylase inhibitor, black = Other mechanism

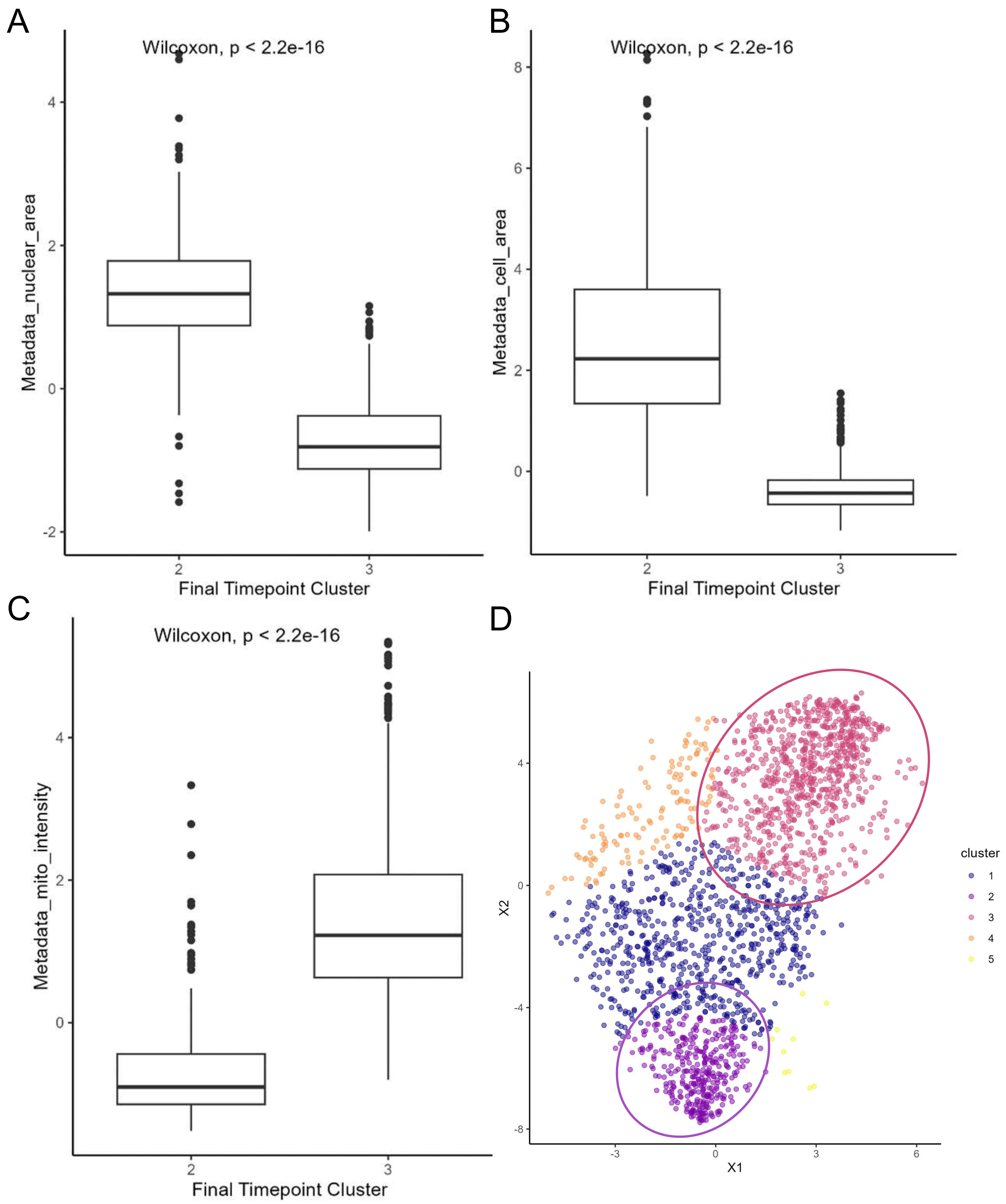

Figure S4. Morphological features used to define HepaRG cell populations, related to Figure 3. Standardised A) nuclear area, B) Cell area and C) Mitotracker intensity for four-week timepoint cells from the two final cell clusters. D) Highlighted trajectory end-point clusters (clusters 2 and 3) from four-week timepoint. Clusters calculated using R package Mclust. Wilcoxon rank sum (Mann-Whitney test) was used after Shapiro-Wilk's normality test. Boxplots represent median with hinges corresponding to first and third quartiles.

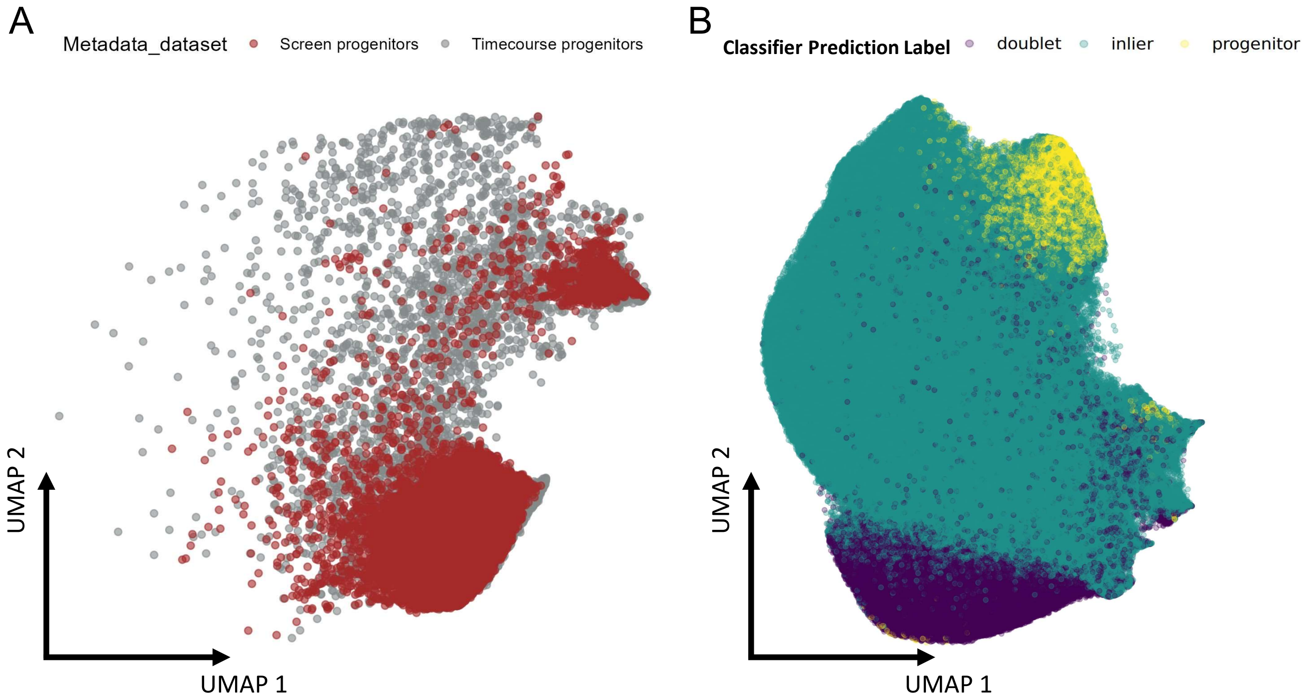

Figure S5. UMAP timecourse dataset overlays, related to Figure 4. A) UMAP overlay of single-cell progenitor Cell Painting data from the timecourse dataset (timepoint 1, 24hrs post seeding) (grey points) and the drug screening dataset (progenitor controls, 24hrs post seeding) (red points). B) UMAP single-cell Cell Painting data coloured by random forest classifier prediction. Predicted doublets (purple) were removed from the dataset.

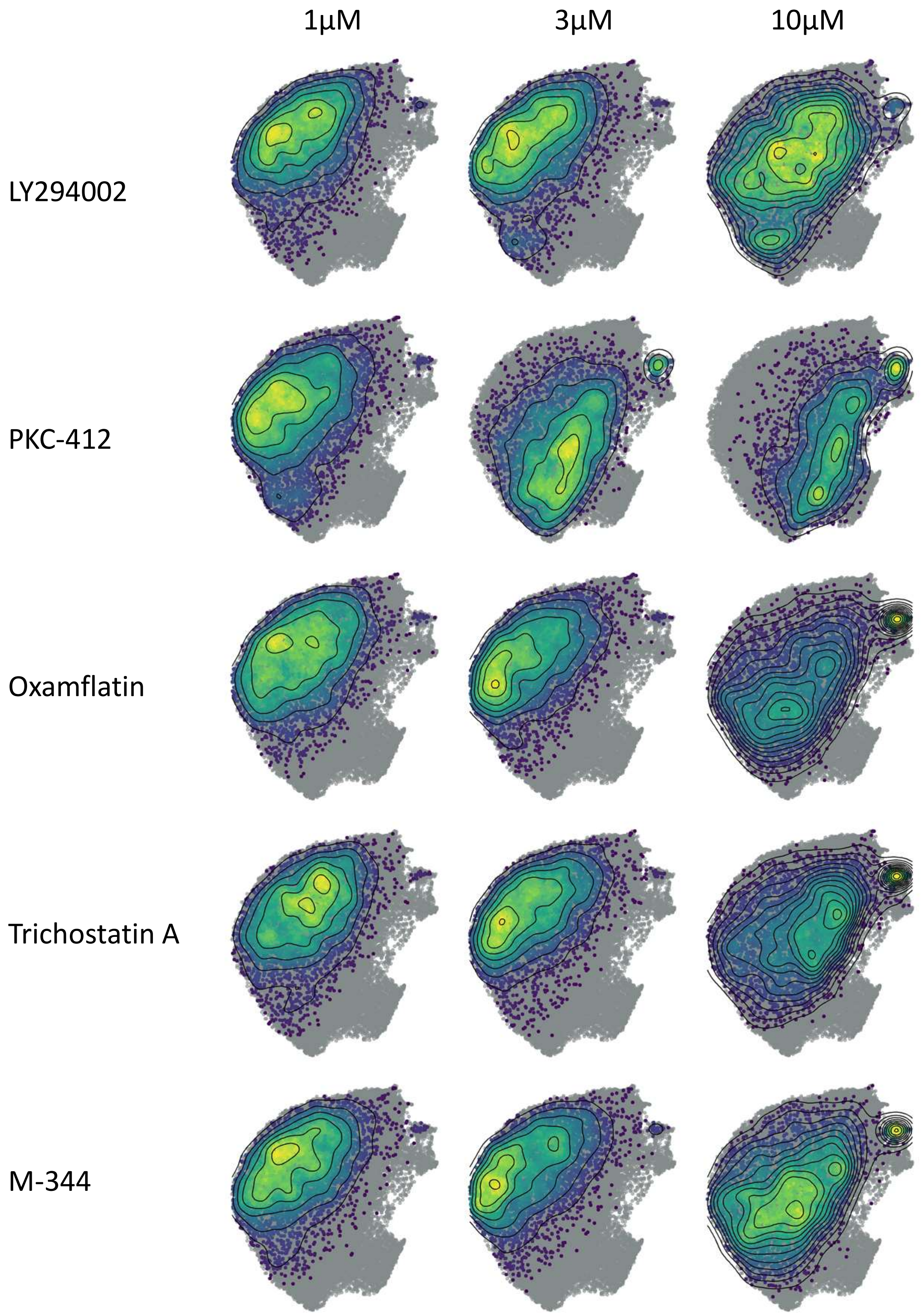

Figure S6. Single cell UMAP overlays for drugs of interest, related to Figure 4. UMAP overlays of HepaRG timecourse (grey) with drug induced single cell data at 1, 3 and 10 μM. Drug single cell data coloured by density of points on plot and density contours added.

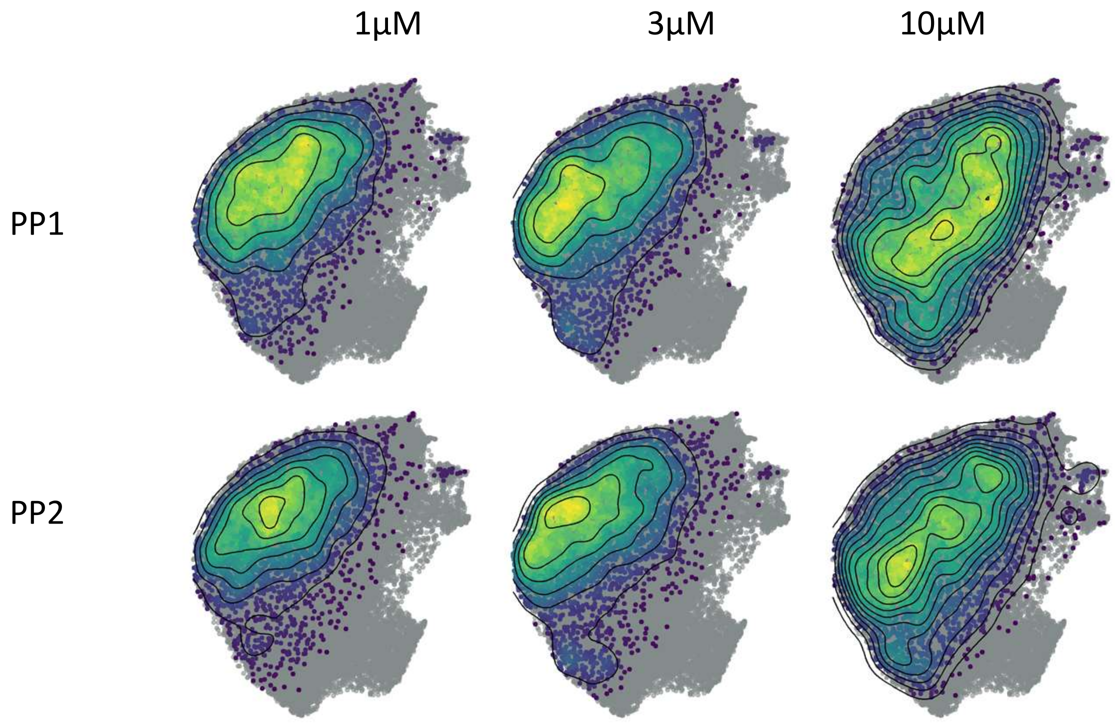

Figure S7. Single cell UMAP overlays for additional SFK inhibitors, related to Figure 4. UMAP overlays of HepaRG timecourse (grey) with drug induced single cell data at 1, 3 and 10 µM. Drug single cell data coloured by density of points on plot and density contours added.

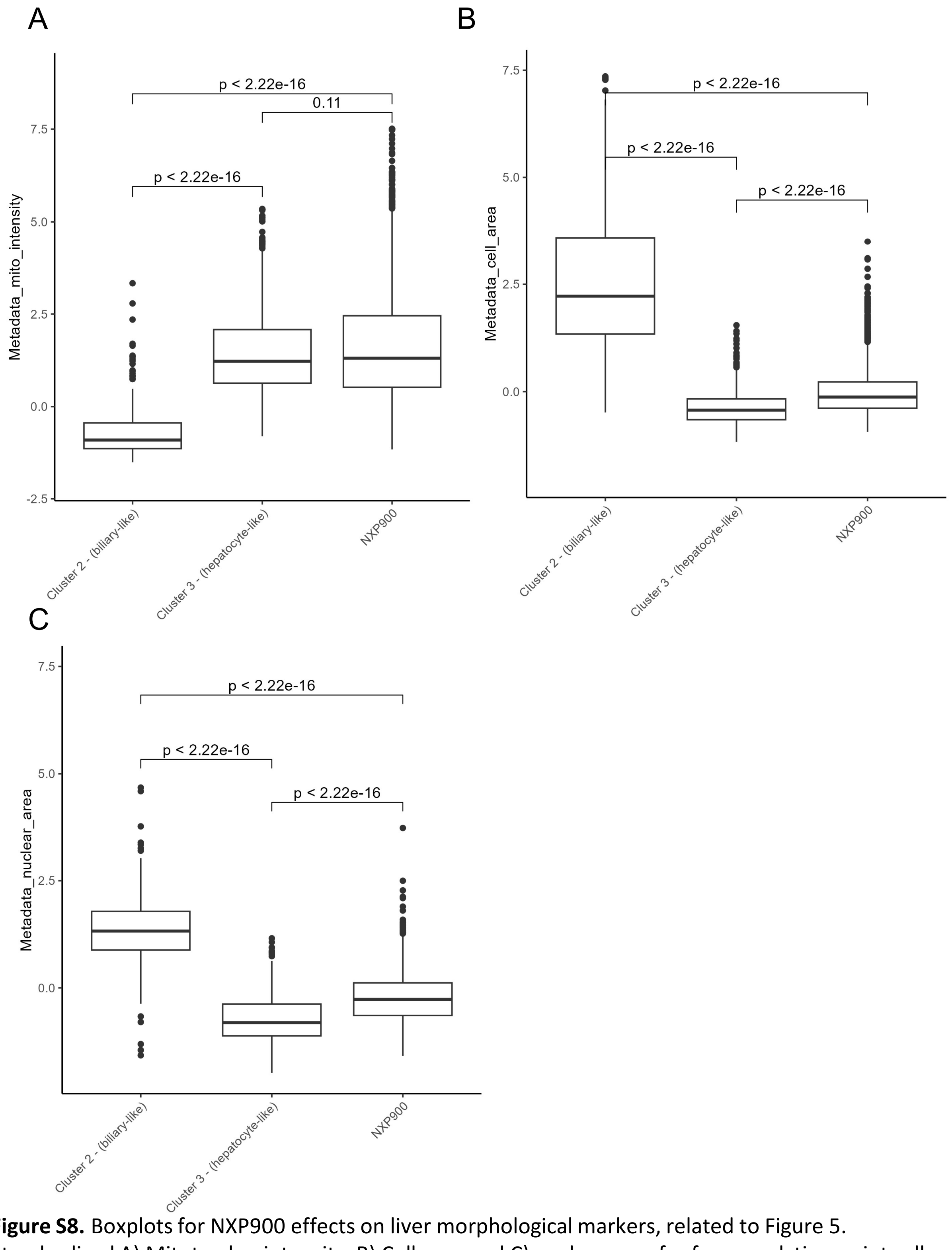

Figure S8. Boxplots for NXP900 effects on liver morphological markers, related to Figure 5. Standardised A) Mitotracker intensity, B) Cell area and C) nuclear area for four-week timepoint cells from the two final cell clusters and day eight NXP900 treatment (10 µM). Wilcoxon rank sum (Mann-Whitney test) was used after Shapiro-Wilk’s normality test. Boxplots represent median with hinges corresponding to first and third quartiles. n = 3

Table S2. Mechanistic class and number of compound hits in class for 27 screening hits, related to Figure 2.

Class	Count
AKT inhibitor	1
Antibiotic	1
Antipsychotic	1
c-Raf Inhibitor	1
Glucocorticoid Antagonist	1
HDAC inhibitor	6
Microtubule Disruptor	4
Mitochondrial Disruptor	2
PI3K inhibitor	3
Protease Inhibitor	1
Proteasome Inhibitor	1
SFK inhibitor	2
Topoisomerase Inhibitor	1
Tyrosine Kinase Inhibitor	2

Table S3. Plate number and days in culture after seeding for timecourse, related to STAR Methods.

Plate Number	Number of days in culture
01	1 day
02	2 days
03	3 days
04	4 days
05	7 days
06	8 days
07	9 days
08	10 days
09	11 days
10	14 days
11	14 days + 1 hr post addition of differentiation supplement
12	14 days + 5.5hrs post addition
13	15 days
14	16 days
15	17 days
16	18 days
17	21 days
18	23 days
19	25 days
20	28 days

Table S4. Cell Painting reagent information, related to STAR Methods**.**

Stain	Structure	Wavelength (ex/em [nm])	Channel	Assay Concentration	Cat No; Supplier
Hoescht 33342	Nuclei	387/447	DAPI	6 µg/mL	#H1399; Mol. Probes
Phalloidin 594	F-actin	562/624	TxRED	0.14X	#ab176757; Abcam
Wheat germ agglutinin Alexa Fluor 594	Golgi and Plasma Membrane	562/624	TxRED	1.5 µg/mL	#W11262; Invitrogen
Concanavalin A Alexa Fluor 488	Endoplasmic Reticulum	462/520	FITC	40 µg/mL	#C11252; Invitrogen
MitoTracker DeepRed	Mitochondria	628/692	CY5	600 nM	#M22426; Invitrogen

Table S5. Compounds and concentrations for primary human hepatic progenitor cell differentiation assay, related to STAR Methods.

Compound	Concentration	Cat No; Supplier
PP1	10 µM	S7060, Selleckchem
PP2	10 µM	S7008, Selleckchem
NXP900	3 µM	S0791, Selleckchem
Vorinostat	5 µM	S1047, Selleckchem
PKC-412	3 µM	10459, Cayman Chemical
Wortmannin	1 µM	S2758, Selleckchem

Table S6. Primary human hepatic progenitor cell assay phenotypic panel information, related to STAR Methods.

Stain	Phenotypic marker	Cell fixation method	Assay Concentration	Cat No; Supplier
CK7	Biliary epithelial cell (HPC)	1:1 methanol acetone	0.36 µg/mL	#ab68459; Abcam
HNF4α	Hepatocyte	10% formalin	3.3 µg/mL	#PP-H1415-0C; R&D Systems
Albumin	Hepatocyte	1:1 methanol acetone	3.3 µg/mL	#ab2406, Abcam
CYP2E1	Hepatocyte	1:1 methanol acetone	0.3 µg/mL	#HPA009128, Atlas Antibodies
Ki67	Proliferation	10% formalin	0.1 µg/mL	#ab1667, Abcam
Cell Mask	Cell membrane	n/a	0.5 µg/mL	#C10046, ThermoFisher
DAPI	nuclei	n/a	1 µg/mL	#D9542, Scientific Laboratory Supplies
